# Immediate Effects of Two Different Methods of Trunk Elastic Taping on Pelvic Inclination, Trunk Impairment, Balance, and Gait in Stroke Patients

**DOI:** 10.3390/medicina60101609

**Published:** 2024-10-01

**Authors:** Eui-Young Jung, Jin-Hwa Jung, Won-Ho Choi

**Affiliations:** 1 Department of Health Science, Gachon University Graduate School, Incheon 21936, Republic of Korea; noel950@gachon.ac.kr; 2 Department of Occupational Therapy, Semyung University, Jecheon 27136, Republic of Korea; otsalt@semyung.ac.kr; 3 Department of Physical Therapy, Gachon University, Incheon 21936, Republic of Korea

**Keywords:** stroke, pelvic tilt, postural balance, gait, trunk impairment

## Abstract

*Background and Objectives*: Stroke patients often experience changes in their pelvic tilt, trunk impairments and decreased gait and balance. While various therapeutic interventions have been attempted to improve these symptoms, there is a need for interventions that are easy to apply and reduce the physical labor of physical and occupational therapists. We aimed to investigate the immediate effects of two different methods of trunk elastic taping on the pelvic inclination, trunk impairment, balance, and gait in chronic stroke patients. *Materials and Methods*: We performed a single-blind randomized controlled trial involving 45 patients with chronic stroke. Participants were randomly assigned to one of three groups: forward rotation with posterior pelvic tilt taping (FRPPT, n = 14), backward rotation with posterior pelvic tilt taping (BRPPT, n = 14), or placebo taping (PT = 14). This study was conducted from December 2023 to January 2024. All the measurements were performed twice: before the intervention and immediately after the intervention. The pelvic inclination was assessed using the anterior pelvic tilt angle. The trunk impairment scale (TIS) was used to measure the trunk impairment. The balance and gait were evaluated using a force plate and walkway system. *Results*: The pelvic inclination was significantly different in the FRPPT and BRPPT groups compared to the PT group (*p* < 0.05, *p* < 0.001). The TIS and gait were significantly increased in the FRPPT group compared to the PT group (*p* < 0.05). The balance significantly improved in the FRPPT and BRPPT within groups (*p* < 0.05). *Conclusions*: Two different methods of posterior pelvic tilt taping improved the anterior pelvic tilt in chronic hemiplegic stroke patients compared with PT, and the FRPPT method also improved the trunk impairment and gait. Therefore, posterior pelvic tilt taping can be used as an intervention with immediate effect.

## 1. Introduction

Stroke is defined as a rapidly developing clinical sign of focal or global disturbance of cerebral function, leading to long-lasting consequences over a lifetime, with no apparent nonvascular cause [1]. Therefore, stroke survivors experience physical changes such as alterations in their pelvic tilt alignment [2] and suffer from decreased trunk function, gait, and balance [3,4,5]. In particular, the pelvis is a key structure in relation to gait, connecting the trunk to the lower extremities, and plays a role in supporting the body and transferring loads to the lower extremities [6]. Patients with stroke have increased postural sway during standing [7] and an abnormal gait [8] due to pelvic instability, which are the major causes of reduced participation and limited activity in patients with chronic stroke [9].

Patients with hemiplegic stroke are characterized by an increased anterior tilt in the sagittal plane and rotation of the pelvis in the horizontal plane compared to healthy adults [10,11]. This anteriorly tilted pelvis contributes to poor trunk support, reduced standing balance, and impairments in the lower extremities [7,12,13]. Furthermore, it can induce changes in gait characteristics, such as decreased symmetry in weight-bearing, step length, stride length, and step width [2,11,14]. Proper pelvic control is critical for economic movement and gait [15]. Therefore, interventions aimed at improving pelvic alignment, enhancing stability, and augmenting gait function are important in clinical practice for patients with stroke who have an unstable pelvis [16,17].

Recently, elastic taping has gained interest as an intervention for musculoskeletal problems, lymphedema, and athletes [18,19,20,21]. It is known that elastic taping can solve patients’ problems by facilitating muscle stimulation through mechanoreceptor stimulation [18,22], promoting proprioception [19], and improving alignment through mechanical force [23]. Recently, interventions using elastic taping are also being performed in stroke patients [24,25,26]. Studies have demonstrated that elastic taping can improve the gait and muscle strength in stroke patients by improving the range of motion and proprioception and reducing spasticity [25,27]. These findings suggest that elastic taping may positively affect patients with stroke. Additionally, studies that applied pelvic taping to subjects with excessive anterior pelvic tilt reported that trunk elastic taping can also improve the pelvic tilt [28,29]. Likewise, various types of taping for pelvic correction applied to stroke patients have been reported to show improvements in the pelvic tilt, gait, balance, and trunk control due to the stimulation of weakened trunk muscle activity and the mechanical force of taping [13,30,31].

There are various taping methods aimed at correcting the anterior pelvic tilt in stroke patients, but the immediate effect of trunk elastic taping is not clear [31,32]. Considering the limited treatment time for chronic stroke patients, studies on the immediate effects of trunk taping are also needed. In several studies, trunk taping has been applied with pelvic backward rotation in the direction of the external oblique abdominis muscle fibers [28,29,33]. Because this application method differs from pelvic frontal rotation, which is recognized as a synergistic movement pattern observed in normal gait, a new directional taping method is needed [34]. Therefore, we aimed to investigate the immediate effects of two different methods of trunk elastic taping on the pelvic inclination, trunk impairment, balance, and gait in chronic hemiplegic stroke.

## 2. Materials and Methods

### 2.1. Study Design

This study was designed as a single-blind randomized controlled trial. To ensure single blinding, the participants were treated one at a time in an independent space such that they were unaware of the group they belonged to or the types of groups in the study. Participants were instructed not to share the interventions with each other. This study was approved by the Gachon University Institutional Review Board (1044396-202311-HR-222-01) and registered with the Clinical Research Information Services (KCT: 0009365). All the participants were recruited through posts from hospitals in Incheon during the month of December 2023. All the participants provided informed consent before participating in the study. All the procedures were conducted in accordance with the principles of the Declaration of Helsinki. 

### 2.2. Participants

This study was conducted at the M Hospital in Incheon, South Korea, from December 2023 to January 2024. Participants were recruited based on the following inclusion criteria: (1) > 6 months poststroke (ischemic or hemorrhage); (2) anterior pelvic tilt > 15°; (3) able to walk at least 15 m independently without assistance; (4) hemiparesis; (5) Brunnstrom recovery stage of the lower extremities ≥ grade 3; (6) Mini-Mental State Examination (MMSE) score ≥ 24; and (7) aged between 30 and 85 years [30,31,33,35]. The exclusion criteria were as follows: (1) vestibular impairment or complaint of dizziness; (2) visual or hearing impairment; (3) neglect or sensory loss; (4) orthopedic conditions of the lower extremities affecting gait; (5) skin allergies to taping; and (6) non-pathological hyperlordosis [31,33]. 

**Figure 1:**
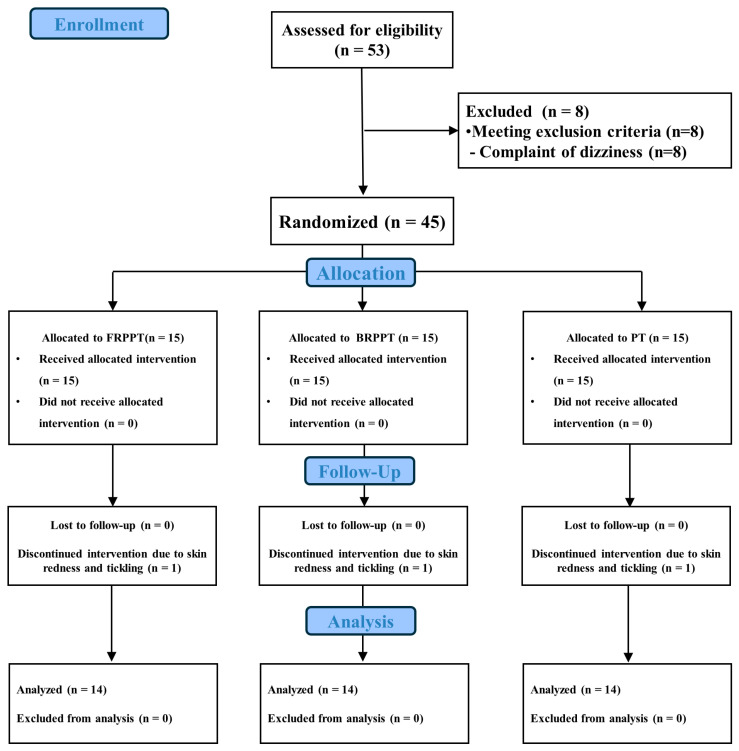
CONSORT flow diagram.

The sample size was calculated using G-Power software version 3.1.9.7 (Hein-rich Heine University, Dusseldorf, North Rhine-Westphalia, Germany). To the best of our knowledge, no study has attempted a similar method, so we used Cohen’s f medium effect size of 0.25 and alpha error probability and power of 0.05 and 0.80, respectively [36]. Consequently, a sample size of 42 was required. To account for an anticipated dropout rate of 20%, 53 participants were enrolled with voluntary consent, and 45 participants participated in the study, after excluding 8 patients who complained of dizziness. The CONSORT flow diagram is shown in Figure 1.

### 2.3. Group Allocation and Randomization

Randomization was performed using codes from a physical therapist who was unaware of the study’s hypothesis and groups. A total of 45 participants were assigned using the stratified randomized permuted block method, with 3 participants set as one block to assign the same number of participants per group using Microsoft Excel (Microsoft Office 365) software. Measurements were taken before and after the intervention. A total of 42 people were evaluated after the intervention, excluding 3 participants who discontinued the intervention because of skin redness and tickling. The evaluation was performed by one physical therapist with >3 years of experience, who did not perform the taping intervention or group allocation.

### 2.4. Intervention

Pelvic taping was performed by a physical therapist with >3 years of experience (Kinesiology 3NS tape, Golden Health Farm, Gimpo, Gyeonggi Province, Republic of Korea), following the guidelines of Dr. Kenzo Kase [37]. All the interventions were performed between 9:00 AM and 5:00 PM. Before applying the elastic tape, the application area was shaved and cleaned.

#### 2.4.1. Forward Rotation with Posterior Pelvic Tilt Taping (FRPPT)

**Figure 2:**
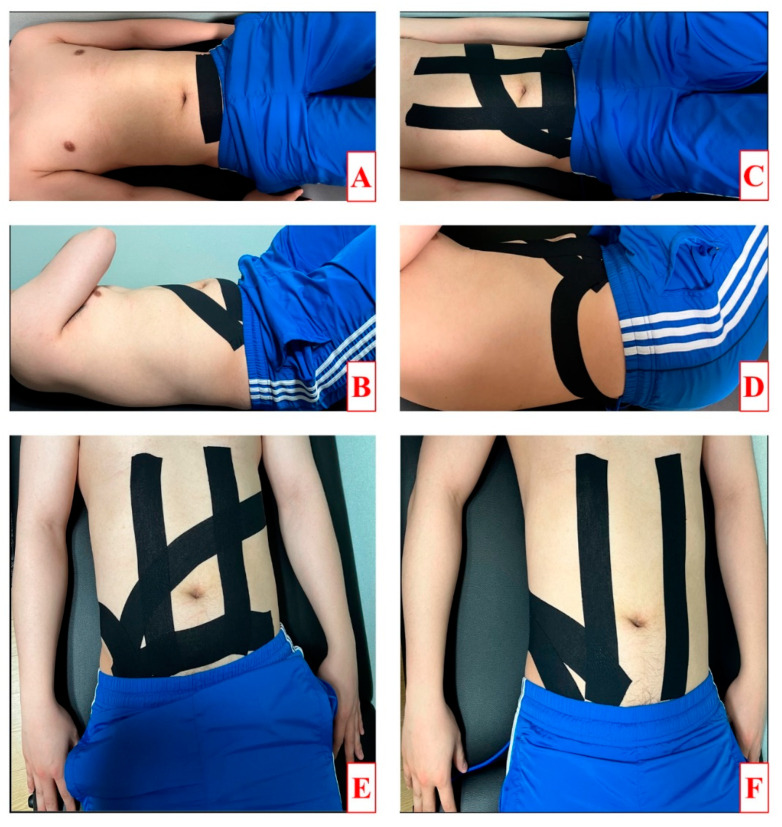
Taping methods. (**A**–**D**) Order of the FRPPT method. (**E**) FRPPT. (**F**) BRPPT.

The FRPPT consisted of three tapings for muscle facilitation and one for mechanical correction, as shown in Figure 2A–E [32,38]. The first tape was applied to the transversus abdominis with a 5 cm wide I-shaped band at 50% tension, from the anterior superior iliac spine (ASIS) on the paretic side to the ASIS on the nonparetic side, with the patient in a hook-lying position. The second taping was performed with the patient in a hook-lying position with the paretic hand on the nonparetic shoulder and the body rotated, with a 5 cm wide, I-shaped band applied to the internal obliques at 50% tension from the ASIS on the paretic side to the outer ribs on the nonparetic side. The third tape was applied to the rectus abdominis from the pubis symphysis to the xiphoid process with a 5 cm wide I-shaped band at 50% tension, with the patient in a hook-lying position. The above three tapes for the Kenzo method were applied in sequence to promote abdominal muscle activation from deep to superficial. The fourth tape was a 5 cm wide, I-shaped band applied at 75% tension from the ASIS to the posterior superior iliac spine (PSIS), with the patient in a side-lying position for pelvic mechanical correction.

#### 2.4.2. Backward Rotation with Posterior Pelvic Tilt Taping (BRPPT)

The BRPPT involved two muscles and one mechanical correction taping, as shown in Figure 2F [22]. The first tape was applied to the external obliques from the inguinal region to the spinous process of the 12th thoracic vertebra in a side-lying position with the knees bent at 90° and a 5 cm wide I-shaped band at 50% tension. The second tape was applied to the rectus abdominis, and the third tape was the pelvic mechanical correction tape.

#### 2.4.3. Placebo Taping (PT)

The PT involved applying a 5 cm wide, I-shaped band 2 cm above the navel without tension, with the patient in a hook-lying position.

### 2.5. Outcome Measures

All the measurements were performed twice: before the intervention and immediately after the intervention.

#### 2.5.1. Pelvic Inclination Angle

**Figure 3:**
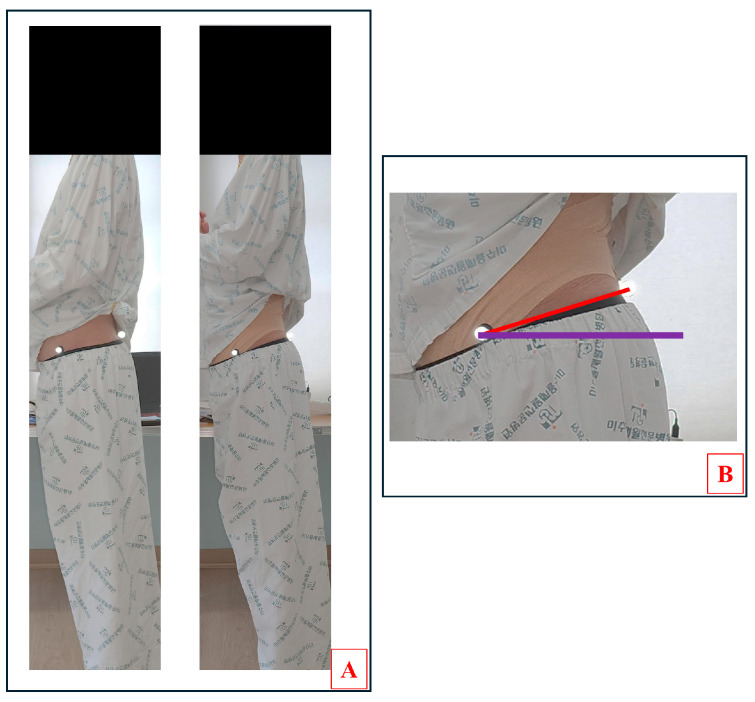
Anterior pelvic tilt angle assessment. (**A**) Pre–post assessments. (**B**) Pelvic angle. The two points are ASIS (left) and PSIS (right). The red line is the connection point of the two points. The purple line is horizontal line.

The pelvic inclination angle was measured as the anterior pelvic tilt angle using a 12-megapixel Samsung camera and 10 mm reflex markers. Participants were instructed to stand inside a 250 cm² square box marked on the floor with arms crossed in front of the chest and a forward gaze. The camera was leveled using a bubble level fixed on a tripod at the height of the patient’s pelvis, and images were captured at a distance of 2 m in a square box. Reflex markers were attached to the participant’s ASIS and PSIS to measure the anterior pelvic tilt angle. Data collected through the camera were measured at angles to the horizon using ImageJ software (ver. 1.54g, National Institutes of Health, Bethesda, MD, USA). The horizontal line was expressed as 0°, the anterior tilt angle was expressed as a positive number, and all the angles were measured thrice [39,40]. The measurement of the pelvic inclination is shown in Figure 3.

#### 2.5.2. Trunk Impairment Function

The trunk impairment function was assessed using the trunk impairment scale (TIS). The TIS is frequently used to evaluate the trunk balance and quality of trunk movement after stroke [41]. The TIS consists of 17 items: 3 for static sitting balance, 10 for dynamic sitting balance, and 4 for core coordination. Scores range from 0 to 23, with higher scores indicating better trunk function. The Korean version of the TIS has an excellent inter-rater intraclass correlation coefficient (ICC (3,1)) = 0.925–0.951 and test–retest ICC (3,1) = 0.805–0.896 [42].

#### 2.5.3. Balance Ability

The balance ability was evaluated using the static balance with the AMTI AccuSway force plate (Advanced Mechanical Technology, Inc., Watertown, MA, USA), which has good reliability (0.70–0.89) [43]. The AccuSway collected, recorded, and analyzed the center of pressure (COP) data using the Balance Clinic software (ver. 1.5.1, AMTI, Watertown, MA, USA). The participant’s sway area, path length, and velocity were collected through the COP data. A high value indicates more fluctuation while maintaining static balance [44]. Participants were instructed to (1) stand on the force plate with feet abducted 30° and heels 9 cm apart; (2) keep the arms crossed and fixed in front of the chest; and (3) look at the sign that was located at eye level at a distance of 1.5 m. The static balance was measured thrice for 20 s and the middle 10 s was used to eliminate the start and end perturbation [45].

#### 2.5.4. Gait Parameter

The gait analysis was performed using the GAITRite walkway system (dimensions: 457.2 cm × 90.2 cm × 0.64 cm; CIR Systems Inc., Franklin, NJ, USA) to evaluate the spatiotemporal parameters, demonstrating good reliability, with the intra-rater reliability ranging from 0.81 to 0.99 and the inter-rater reliability from 0.77 to 0.99 [46]. To exclude the initial acceleration and terminal deceleration from the results, the start and stop lines were set up 2 m from the walkway, and the patients were instructed to start at the signal and walk at their usual walking speed. Data collected using the walkway were processed using the GAITRite Platinum software (ver. 4.7.7). Spatiotemporal gait parameters, including the velocity, cadence, step length, stride length, and single-support time, were collected. The spatial and temporal symmetries were calculated as follows [47]:

$$Temporal\;symmetry\;ratio=\left|1-\frac{Step\;Length\;\left(affected\right)}{Step\;Length\;\left(unaffected\right)}\right|$$

$$Spatial\;symmetry\;ratio=\left|1-\frac{Single\;Spport\;Time\;\left(affected\right)}{Single\;Spport\;Time\;\left(unaffected\right)}\right|$$

In this equation, “affected” represents the paretic side lower extremity and “unaffected” means the nonparetic side lower extremity. A larger ratio indicates greater asymmetry.

### 2.6. Statistical Analysis

All the statistical analyses were performed using the SPSS statistical software (version 25.0; SPSS Inc., Chicago, IL, USA). The Shapiro–Wilk test was used for the normality test. The participants’ general characteristics were compared using the chi-square test and one-way analysis of variance; 3-by-2 repeated measures analysis of variance (group [FRPPT, BRPPT, PT] by time [preintervention, postintervention]) was conducted to compare the variables. The significance level was corrected using the Bonferroni method. When the interaction was not significant, the main effects of the time and group were evaluated. The significance level was set at α = 0.05. 

## 3. Results

### 3.1. Participants’ General Characteristics

The general characteristics of the participants in each group are presented in Table 1. No significant differences were observed in the baseline general characteristics (*p* > 0.05).

### 3.2. Pelvic Inclination

The anterior pelvic tilt angles are shown in Table 2. No significant difference was noted at baseline. A significant difference between the group and time interaction effect for the pelvic inclination was observed (*p* < 0.001, η^2^_p_ = 0.36). The PT group differed significantly from the FRPPT and BRPPT groups (*p* < 0.05, *p* < 0.001). Additionally, the FRPPT and BRPPT groups showed significant within group differences (*p* < 0.001 and *p* < 0.001, respectively).

### 3.3. Trunk Impairment

The TIS scores are presented in Table 3. A significant difference was observed between the group and time interaction for the TIS and dynamic sitting balance (*p* < 0.001, η^2^_p_ =0.44; *p* = 0.001, η^2^_p_ = 0.316). the BRPPT group had a significant within group improvement in the TIS and dynamic sitting balance (*p* = 0.005, *p* = 0.008), whereas the FRPPT group had a significant within group improvement in the TIS, dynamic sitting balance, and coordination (*p* < 0.001, *p* < 0.001, *p* = 0.009). In addition, the FRPPT group had a significant difference compared to the PT group in the dynamic sitting balance (*p* = 0.022).

### 3.4. Balance

The static balance data are shown in Table 4. No significant difference was noted between the group and time interaction effects for balance. The FRPPT and BRPPT groups showed statistically significant time effects (*p* = 0.021 and *p* = 0.041, respectively).

#### 3.4.1. Gait

The gait parameters are listed in Table 5. Statistically significant group and time interactions were observed for the step length (affected and unaffected), stride length (affected), and single support time. The BRPPT group showed significant within group differences in the velocity, cadence, step length (unaffected), and stride length (unaffected). The FRPPT group showed significant within group improvements in the velocity, cadence, step length (affected and unaffected), stride length (affected and unaffected), and single-support time (affected). The FRPPT group was significantly different from the PT group in terms of the step length (affected and unaffected). 

#### 3.4.2. Gait Symmetry

The gait symmetry parameters are presented in Table 6. No differences were observed in the group and time interaction effects on gait symmetry. The spatial and temporal symmetry significantly only improved in the FRPPT group (*p* < 0.05).

## 4. Discussion

The taping method in this study showed immediate effects on the pelvic inclination, trunk impairment, and gait in chronic stroke patients. First, the anterior pelvic tilt angles in the FRPPT and BRPPT groups were significantly lower than that in the PT group. Elastic taping applies different elasticities depending on the purpose [48,49], and it is known to have various effects, such as improving blood flow and lymphatic fluid circulation through a lifting effect [50], facilitating muscle activation through stimulation of the mechanoreceptor [18,22], improving proprioception [19], and correcting alignment through mechanical force [23]. Several studies have reported improvements in the pelvic tilt angle with taping [28,29,30,32,51,52]. Tahmasbi et al. reported an improvement in the anterior pelvic tilt and lumbar lordosis by applying tape to patients with anterior pelvic tilt, and they argued that this provides stimulation to the mechanoreceptor, promoting contraction of the abdominal muscles, ultimately causing posterior pelvic tilt [51]. Bozorgmehr et al. showed that posterior tilt taping applied to the abdominal muscles for one week increased the thickness of the abdominal muscles until one week after the tape was removed, suggesting that elastic taping may also affect the thickness of the abdominal muscles [52]. Therefore, the taping method used in this study can correct the pelvic tilt by facilitating abdominal muscle activity. Posterior tilt taping is typically applied to the external oblique and rectus abdominis muscles. 

However, the FRPPT in this study was confirmed to modify the pelvic tilt angle even though it was applied to the internal oblique and transversus abdominis instead of the external oblique. This appears to be related to the direction and attachment location of the posterior pelvic tilt tape. Elastic taping stimulates the same mechanoreceptors in the abdominal muscles in similar positions, causing posterior pelvic tilt, although the direction of the taping is different. In addition, the high tension of the tape, which used the mechanical force applied to the pelvis in this study, may have also contributed to the improvement. The high-tension elastic tape used in this study suppressed excessive anterior pelvic tilt and helped activate the abdominal muscles by optimizing their length–tension relationship. This activation of the abdominal muscles seems to have contributed to the improvement of the pelvic tilt. Tahmasbi et al. observed changes in the pelvic tilt by varying the tension of the tape (100%, 115%, 140%) on the external oblique and rectus abdominis muscles, reporting that greater tape tension resulted in better pelvic tilt correction [51]. Tanoori et al. also suggested that elastic taping can improve muscle strength by optimizing the length–tension relationship [53]. In this study, the anterior pelvic tilt angle was improved in both the FRPPT and BRPPT groups by the tape attached to the abdominal muscles, suggesting that the anterior pelvic tilt may be improved by mechanical stimulation of the skin through taping and the activation effect of the abdominal muscles through improvement of the length–tension relationship. Furthermore, considering that the pelvic tilt of the recruited stroke patients with excessive anterior pelvic tilt changed to a normal pelvic tilt angle range reported as 11 ± 4° [54] immediately after taping, the results of this study are clinically meaningful. 

In this study, pelvic taping had a positive effect on balance and trunk impairment. Other studies have reported similar results [31,52]. In this study, the group whose TIS scores increased also showed within group improvement in the standing balance. This result is consistent with those reported by Verheyden et al., who showed a strong correlation between the TIS score and balance in patients with stroke [55]. Additionally, the changed pelvic inclination in patients with stroke is closely correlated with the functional ability, gait, and balance [2,11,56,57]. The patients recruited in this study also had decreased trunk function and balance due to the excessive anterior pelvic tilt. Pelvic control is important for trunk stability [58], and a lack of trunk stability can lead to decreased trunk function, decreased balance, and postural imbalance [2,11]. Wang et al. reported improvements in balance and gait stability by evaluating the Berg Balance Scale (BBS) and applying non-elastic taping to the hip [59]. Therefore, increased stability in the trunk may influence the improvement in balance. In this study, correction of the pelvic tilt through elastic taping increased the trunk stability, which may have affected the trunk impairment and balance. However, in this study, balance improvements were only observed within groups, which may be related to the duration of the taping intervention [31,59]. Additionally, the FRPPT group showed differences in the dynamic sitting balance and coordination, which are subscales of the TIS. These results may be related to the ability of the two subscales in the horizontal and lateral planes [55]. In the FRPPT group, the direction of taping applied to induce forward rotation of the pelvis along the internal oblique was consistent with the direction that functionally assists the lateral flexion and rotation of the trunk. Therefore, this study showed that the FRPPT group had different results from the other taping methods in terms of the subscale scores.

Patients with chronic stroke have postural characteristics such as forward leaning posture and anterior pelvic tilt [58], and their gait characteristics include slow gait speed, reduced stride length, step length, and stance time [57]. Several studies have reported that the excessive anterior pelvic tilt in patients with stroke is highly correlated with the gait velocity, cadence, step length, and weight-bearing asymmetry [2,11,14]. Patterson et al. suggested that the decrease in stance time in patients with stroke was related to balance control problems during the stance phase on the paralyzed side [60]. Therefore, an uncontrolled pelvis may cause a loss of balance during a single stance on the paretic leg, resulting in a decrease in weight-bearing ability and stance time, which also appears to result in a decrease in the step and stride length of the nonparetic leg. Shin et al. showed that posterior pelvic tilt taping improved the velocity, step and stride length and pelvic tilt [30]. In et al. reported that 6 weeks of exercise and posterior pelvic tilt taping showed improved pelvic tilt and gait speed due to improvements in the pelvic tilt and an increase in the hip extensor strength [61]. In this study, the improvement in the anterior pelvic tilt due to elastic taping contributed to pelvic stability and ultimately improved gait. Additionally, the gait symmetry and affected side gait parameters showed a significant increase within the group in only the FRPPT group, not in the other groups. Patterson et al. observed temporal asymmetry in approximately 55% of patients with stroke and claimed that this was due to an increased paretic swing and stance duration [62]. Several studies have suggested that the gait parameter on the affected side is reduced due to a decrease in the propulsive force of the affected leg in patients with stroke and that propulsive force can be corrected by controlling trunk progression in such patients [63,64]. Therefore, the FRPPT group achieved stability and mobility on the affected side during gait due to the direction of the anterior pelvic rotation taping, which aligned with the coordinated movement of the pelvis on the affected side. This may have ultimately affected their gait parameters. However, it is unclear whether the taping method used in this study is directly related to the corrected pelvic angle and gait parameters of patients with stroke. 

In this study, we confirmed the effects of three different taping methods on the pelvic inclination, trunk impairment, balance, and gait in patients with chronic hemiplegic stroke. The anterior pelvic tilt, which is commonly observed in such patients, negatively affects the postural control, function, balance, and gait. However, the intervention process is not easy because several factors need to be considered. This study used pelvic angles in the sagittal plane to suggest pelvic instability. It may not be difficult to determine whether people have these characteristics from a single assessment. Therefore, future studies should conduct various evaluations to specify instability, which may help in identifying the exact characteristics of stroke patients. The taping methods presented in this study are effective and offer several advantages. Therefore, they can be applied for short-term effects with the cost–benefit considerations in clinical practice. However, the small number of participants with limited types and a large age range makes it difficult to generalize these results, and the short application time did not allow us to confirm the duration of the effect. Additionally, we were unable to confirm exactly which mechanism our taping affected in terms of each variable, and we could not specifically determine how much it influenced the forward rotation or muscle activity. Therefore, in future studies, it will be necessary to conduct motion analysis with a larger number of subjects to confirm whether taping affects the pelvic tilt and gait in various planes during walking.

## 5. Conclusions

Our study demonstrated that two different methods of posterior pelvic tilt taping improve the anterior pelvic tilt in patients with chronic hemiplegic stroke. In particular, the new FRPPT method effectively improved the trunk impairment and gait. In clinical practice, posterior pelvic taping can be used as an effective intervention with immediate effects and cost–benefit suitability in patients with stroke who have excessive anterior tilt.

## Figures and Tables

**Table 1:** The general characteristics of the participants.

Variables	FRPPT (n = 14)	BRPPT (n = 14)	PT (n = 14)	*p*
Age (years)	63.76 ± 12.66 ^1^	60.41 ± 11.04 ^1^	62.92 ± 9.68 ^1^	0.743 ^3^
Height (cm)	167.00 ± 6.63 ^1^	164.08 ± 8.22 ^1^	166.38 ± 6.73 ^1^	0.575 ^3^
Weight (kg)	64.86 ± 10.67 ^1^	63.73 ± 7.77 ^1^	64.05 ± 9.10 ^1^	0.927 ^3^
Sex (male/female)	9/5	5/9	8/6	0.289 ^2^
Paretic side (right/left)	8/6	6/8	8/6	0.683 ^2^
Stroke type (ischemic/hemorrhage)	9/5	5/9	9/5	0.215 ^2^
Stroke duration (months)	11.07 ± 2.87 ^1^	11.91 ± 2.90 ^1^	10.76 ± 2.08 ^1^	0.238 ^3^
MMSE (score)	26.30 ± 1.70 ^1^	26.66 ± 1.82 ^1^	26.53 ± 1.66 ^1^	0.788 ^3^

^1^ mean ± standard deviation, ^2^ chi-square test, ^3^ one-way analysis of variance (ANOVA). MMSE, Mini-Mental State Examination.

**Table 2:** Pelvic inclination.

Variables	Pre 95% CI [Low–High]	Post 95% CI [Low–High]	Within Group *p*-Value	Between Groups *p*-Value	Group × Time *p*-Value	F	η^2^_p_
Anterior pelvic tilt (degree)							
FRPPT	16.51 ± 1.06 [15.89–17.14]	12.99 ± 3.25 ^†^ [11.27–14.73]	<0.001	0.006	<0.001	10.043	0.36
BRPPT	16.91 ± 1.32 [16.26–17.57]	12.12 ± 3.04 ^††^ [10.33–13.92]	<0.001				
PT	16.25 ± 0.92 [15.63–16.88]	16.11 ± 2.88 [14.39–17.84]	0.853				

Data are expressed as the mean ± standard deviation. All the baselines showed no significant difference. BRPPT, backward rotation with posterior pelvic tilt taping; FRPPT, forward rotation with posterior pelvic tilt taping; PT, placebo taping; η*^2^*_p_, partial eta squared. ^†^ Significant difference compared to the placebo taping group, ^†^ = *p* < 0.05, ^††^ = *p* < 0.001.

**Table 3:** Trunk impairment.

Variables	Pre 95% CI [Low–High]	Post 95% CI [Low–High]	Within Group *p*-Value	Between Groups *p*-Value	Group × Time *p*-Value	F	η^2^_p_
Trunk impairment scale (score)							
FRPPT	14.76 ± 0.75 [13.24–16.30]	16.61 ± 0.78 [15.03–18.20]	< 0.001	0.208	<0.001	13.767	0.44
BRPPT	14.83 ± 0.78 [13.24–16.43]	15.66 ± 0.81 [14.02–17.32]	0.005				
PT	14.76 ± 0.75 [13.24–16.30]	14.61 ± 0.78 [13.03–16.20]	0.572				
Static sitting balance (score)							
FRPPT	6.69 ± 0.48 [6.12–7.27]	6.84 ± 0.37 [6.27–7.42]	0.050	0.143	0.366	1.034	0.056
BRPPT	6.08 ± 1.72 [5.48–6.68]	6.16 ± 1.74 [5.57–6.76]	0.298				
PT	6.92 ± 0.27 [6.35–7.50]	6.92 ± 0.27 [6.35–7.50]	1.000				
Dynamic sitting balance (score)							
FRPPT	5.84 ± 2.33 [4.78–6.91]	7.23 ± 2.24 ^†^ [6.19–8.28]	<0.001	0.205	0.001	8.076	0.316
BRPPT	5.91 ±1.88 [4.81–7.02]	6.66 ± 1.82 [5.58–7.76]	0.008				
PT	5.02 ± 1.30 [4.17–6.30]	5.15 ± 1.40 [4.11–6.20]	0.767				
Coordination (score)							
FRPPT	2.23 ± 0.83 [1.61–2.86]	2.53 ± 0.96 [1.87–3.21]	0.009	0.781	0.049	3.292	0.158
BRPPT	2.83 ± 1.33 [2.18–3.48]	2.83 ± 1.33 [2.13–3.54]	1.000				
PT	2.61 ± 1.10 [1.99–3.24]	2.53 ± 1.26 [1.87–3.21]	0.496				

Data are expressed as the mean ± standard deviation. All the baselines showed no significant difference. BRPPT, backward rotation with posterior pelvic tilt taping; FRPPT, forward rotation with posterior tilt taping; PT, placebo taping; η^2^_p_, partial eta squared. ^†^ Significant difference compared to the placebo taping group, ^†^ = *p* < 0.05.

**Table 4:** Static balance.

Variables	Pre 95% CI [Low–High]	Post 95% CI [Low–High]	Within Group *p*-Value	Between Groups *p*-Value	Group × Time *p*-Value	F	η^2^_p_
Sway area (mm^2^)							
FRPPT	4.47 ± 2.23 [3.16–5.77]	3.51 ± 1.55 [2.48–4.54]	0.021	0.205	0.057	3.123	0.15
BRPPT	3.77 ± 2.31 [2.40–5.15]	2.89 ± 0.87 [1.83–3.97]	0.041				
PT	3.92 ± 2.47 [2.61–5.25]	4.23 ± 2.57 [3.20–5.26]	0.453				
Sway path length (mm)							
FRPPT	22.09 ± 8.67 [17.89–26.29]	20.71 ± 8.10 [17.02–24.42]	0.139	0.517	0.847	0.167	0.009
BRPPT	19.26 ± 6.59 [14.90–23.64]	17.73 ± 5.57 [13.89–21.58]	0.113				
PT	20.63 ± 6.88 [16.43–24.83]	19.81 ± 5.60 [16.12–23.51]	0.373				
Sway velocity (mm/s)							
FRPPT	2.20 ± 0.86 [1.79–2.62]	2.07 ± 0.81 [1.70–2.44]	0.141	0.517	0.780	0.250	0.014
BRPPT	1.94 ± 0.63 [1.51–2.38]	1.77 ± 0.55 [1.39–2.16]	0.075				
PT	2.07 ± 0.72 [1.65–2.48]	1.98 ± 0.56 [1.61–2.35]	0.376				

Data are expressed as the mean ± standard deviation. All the baselines showed no significant difference. BRPPT, backward rotation with posterior pelvic tilt taping; FRPPT, forward rotation with posterior pelvic tilt taping; PT, placebo taping; η^2^p, partial eta squared.

**Table 5:** Gait.

Variables	Pre 95% CI [Low–High]	Post 95% CI [Low–High]	Within Group *p*-Value	Between Groups *p*-Value	Group × Time *p*-Value	F	η^2^_p_
Velocity (cm/s)							
FRPPT	51.75 ± 15.09 [42.73–60.77]	59.22 ± 17.18 [49.14–69.31]	<0.001	0.323	0.024	4.152	0.192
BRPPT	48.81 ± 19.38 [39.42–58.20]	53.77 ± 22.56 [43.28–64.27]	0.009				
PT	47.92 ± 13.27 [38.91–56.95]	48.49 ± 13.16 [38.42–58.58]	0.741				
Cadence (steps/min)							
FRPPT	74.18 ± 18.26 [68.17–85.20]	78.59 ± 18.17 [67.16–90.03]	0.009	0.278	0.081	2.707	0.134
BRPPT	70.67 ± 21.86 [59.20–82.15]	74.19 ± 23.18 [62.29–86.10]	0.040				
PT	71.40 ± 18.58 [60.39–82.42]	70.90 ± 19.49 [59.47–82.34]	0.756				
Step length—affected (cm)							
FRPPT	37.22 ± 7.32 [32.13–42.31]	43.04 ± 8.31 ^†^ [37.83–48.25]	<0.001	0.026	<0.001	9.835	0.360
BRPPT	34.40 ± 11.56 [29.11–39.70]	36.04 ± 12.28 [30.61–41.47]	0.151				
PT	33.72 ± 7.86 [28.64–38.82]	32.91 ± 6.51 [27.70–38.12]	0.450				
Step length—unaffected (cm)							
FRPPT	36.56 ± 3.13 [30.20–42.96]	42.19 ± 3.11 ^†^ [35.87–48.52]	<0.001	0.031	<0.001	7.966	0.313
BRPPT	32.93 ± 3.26 [26.31–39.56]]	35.78 ± 3.24 [29.20–42.38]	0.040				
PT	31.56 ± 3.13 [25.20–37.92]	30.01 ± 3.11 [23.69–36.34]	0.236				
Stride length—affected (cm)							
FRPPT	69.82 ± 12.98 [59.28–80.37]	77.02 ± 14.20 [66.16–87.88]	<0.001	0.298	0.005	6.186	0.261
BRPPT	65.31 ± 21.70 [54.34–76.30]	68.19 ± 22.76 [56.89–79.50]	0.070				
PT	65.66 ± 20.57 [55.12–76.22]	65.55 ± 20.20 [54.70–76.49]	0.940				
Stride length—unaffected (cm)							
FRPPT	68.48 ± 11.94 [57.87–79.10]	74.42 ± 14.30 [63.44–85.41]	0.001	0.519	0.141	2.076	0.106
BRPPT	64.95 ± 21.94 [53.91–76.00]	68.55 ± 22.85 [57.12–79.95]	0.036				
PT	64.38 ± 21.27 [53.77–75.00]	65.75 ± 20.66 [54.76–76.74]	0.396				
Single support time—affected (second)							
FRPPT	0.39 ± 0.05 [0.35–0.43]	0.42 ± 0.04 [0.39–0.46]	0.011	0.175	0.022	4.291	0.197
BRPPT	0.37 ± 0.07 [0.33–0.41]	0.39 ± 0.06 [0.35–0.43]	0.155				
PT	0.39 ± 0.07 [0.34–0.42]	0.38 ± 0.06 [0.33–0.41]	0.185				

Data are expressed as the mean ± standard deviation. All the baselines showed no significant difference. BRPPT, backward rotation with posterior pelvic tilt taping; FRPPT, forward rotation with posterior pelvic tilt taping; PT, placebo taping; η^2^_p_, partial eta squared. ^†^ Significant difference compared to the placebo taping group, ^†^ = *p* < 0.05.

**Table 6:** Gait symmetry.

Variables	Pre 95% CI [Low–High]	Post 95% CI [Low–High]	Within Group *p*-Value	Between Groups *p*-Value	Group × Time *p*-Value	F	η^2^_p_
Spatial symmetry (ratio)							
FRPPT	0.14 ± 0.14 [0.08–0.21]	0.07 ± 0.08 [0.02–0.13]	0.003	0.185	0.158	1.944	0.100
BRPPT	0.15 ± 0.11 [0.08–0.22]	0.11 ± 0.09 [0.06–0.17]	0.113				
PT	0.15 ± 0.08 [0.09–0.22]	0.14 ± 0.11 [0.09–0.20]	0.666				
Temporal symmetry (ratio)							
FRPPT	0.15 ± 0.07 [0.09–0.21]	0.08 ± 0.05 [0.03–0.14]	0.005	0.165	0.062	3.010	0.147
BRPPT	0.15 ± 0.13 [0.10–0.21]	0.12 ± 0.13 [0.07–0.19]	0.321				
PT	0.15 ± 0.06 [0.09–0.21]	0.15 ± 0.09 [0.10–0.22]	0.665				

BRPPT, backward rotation with posterior pelvic tilt taping; FRPPT, forward rotation with posterior pelvic tilt taping; PT, placebo taping; η^2^_p_, partial eta squared.

## Data Availability

The data for this study are available from the corresponding authors on reasonable request.

## References

[1] Investigators W.M.P.P. (1988). The World Health Organization MONICA Project (monitoring trends and determinants in cardiovascular disease): A major international collaboration. J. Clin. Epidemiol..

[2] Karthikbabu S., Chakrapani M., Ganesan S., Ellajosyula R. (2016). Relationship between pelvic alignment and weight-bearing asymmetry in community-dwelling chronic stroke survivors. J. Neurosci. Rural. Pract..

[3] Bohannon R.W., Cassidy D., Walsh S. (1995). Trunk muscle strength is impaired multidirectionally after stroke. Clin. Rehabil..

[4] Heshmatollah A., Darweesh S.K., Dommershuijsen L.J., Koudstaal P.J., Ikram M.A., Ikram M.K. (2020). Quantitative gait impairments in patients with stroke or transient ischemic attack: A population-based approach. Stroke.

[5] Chern J.-S., Lo C.-Y., Wu C.-Y., Chen C.-L., Yang S., Tang F.-T. (2010). Dynamic postural control during trunk bending and reaching in healthy adults and stroke patients. Am. J. Phys. Med. Rehabil..

[6] Dubey L., Karthikbabu S., Mohan D. (2018). Effects of pelvic stability training on movement control, hip muscles strength, walking speed and daily activities after stroke: A randomized controlled trial. Ann. Neurosci..

[7] Dickstein R., Abulaffio N. (2000). Postural sway of the affected and nonaffected pelvis and leg in stance of hemiparetic patients. Arch. Phys. Med. Rehabil..

[8] Kerrigan D.C., Frates E.P., Rogan S., Riley P.O. (2000). Hip hiking and circumduction: Quantitative definitions. Am. J. Phys. Med. Rehabil..

[9] Andrenelli E., Ippoliti E., Coccia M., Millevolte M., Cicconi B., Latini L., Lagalla G., Provinciali L., Ceravolo M.G., Capecci M. (2015). Features and predictors of activity limitations and participation restriction 2 years after intensive rehabilitation following first-ever stroke. Eur. J. Phys. Rehabil. Med..

[10] Kim H.-S., Chung S.-C., Choi M.-H., Gim S.-Y., Kim W.-R., Tack G.-R., Lim D.-W., Chun S.-K., Kim J.-W., Mun K.-R. (2016). Primary and secondary gait deviations of stroke survivors and their association with gait performance. J. Phys. Ther. Sci..

[11] Kim M.-K., Kim S.-G., Shin Y.-J., Choi E.-H., Choe Y.-W. (2017). The relationship between anterior pelvic tilt and gait, balance in patient with chronic stroke. J. Phys. Ther. Sci..

[12] Karthikbabu S., Chakrapani M., Ganesan S., Ellajosyla R. (2017). Pelvic alignment in standing, and its relationship with trunk control and motor recovery of lower limb after stroke. Neurol. Clin. Neurosci..

[13] Mehta M., Joshua A.M., Karthikbabu S., Misri Z., Unnikrishnan B., Mithra P., Nayak A. (2019). Effect of taping of thoracic and abdominal muscles on pelvic alignment and forward reach distance among stroke subjects: A randomized controlled trial. Ann. Neurosci..

[14] Gurli H., Ganvir S. (2019). Effect of pelvic tilt on gait parameters in patients with stroke: Effect of pelvic tilt on parameters of gait in stroke patients. VIMS J. Phys. Ther..

[15] Staszkiewicz R., Chwała W., Forczek W., Laska J. (2012). Three-dimensional analysis of the pelvic and hip mobility during gait on a treadmill and on the ground. Acta. Bioeng. Biomech..

[16] Kooncumchoo P., Namdaeng P., Hanmanop S., Rungroungdouyboon B., Klarod K., Kiatkulanusorn S., Luangpon N. (2021). Gait improvement in chronic stroke survivors by using an innovative gait training machine: A randomized controlled trial. Int. J. Environ. Res. Public Health.

[17] Gama G.L., Celestino M.L., Barela J.A., Forrester L., Whitall J., Barela A.M. (2017). Effects of gait training with body weight support on a treadmill versus overground in individuals with stroke. Arch. Phys. Med. Rehabil..

[18] Vithoulka I., Beneka A., Malliou P., Aggelousis N., Karatsolis K., Diamantopoulos K. (2010). The effects of Kinesio-Taping® on quadriceps strength during isokinetic exercise in healthy non athlete women. Isokinet. Exerc. Sci..

[19] Bischoff L., Babisch C., Babisch J., Layher F., Sander K., Matziolis G., Pietsch S., Röhner E. (2018). Effects on proprioception by Kinesio taping of the knee after anterior cruciate ligament rupture. Eur. J. Orthop. Surg. Traumatol..

[20] Marotta N., Lippi L., Ammendolia V., Calafiore D., Inzitari M.T., Pinto M., Invernizzi M. (2023). Efficacy of kinesio taping on upper limb volume reduction in patients with breast cancer-related lymphedema: A systematic review of randomized controlled trials. Eur. J. Phys. Rehabil. Med..

[21] Reneker J.C., Latham L., McGlawn R., Reneker M.R. (2018). Effectiveness of kinesiology tape on sports performance abilities in athletes: A systematic review. Phys. Ther. Sport.

[22] Konishi Y. (2013). Tactile stimulation with Kinesiology tape alleviates muscle weakness attributable to attenuation of Ia afferents. J. Sci. Med. Sport.

[23] Yang S.R., Heo S.Y., Lee H.J. (2015). Immediate effects of kinesio taping on fixed postural alignment and foot balance in stroke patients. J. Phys. Ther. Sci..

[24] Bae Y., Park D. (2022). Immediate effect of lower-leg kinesio taping on ankle dorsiflexion and gait parameters in chronic Stroke with foot drop. J. Stroke Cerebrovasc. Dis..

[25] Rojhani-Shirazi Z., Amirian S., Meftahi N. (2015). Effects of ankle kinesio taping on postural control in stroke patients. J. Stroke Cerebrovasc. Dis..

[26] Hu Y., Zhong D., Xiao Q., Chen Q., Li J., Jin R. (2019). Kinesio taping for balance function after stroke: A systematic review and meta-analysis. Evid.-Based Complement. Altern. Med..

[27] In T.-S., Jung J.-H., Jung K.-S., Cho H.-Y. (2021). Effectiveness of transcutaneous electrical nerve stimulation with taping for stroke rehabilitation. BioMed Res. Int..

[28] Lee J.-H., Yoo W.-G. (2012). Application of posterior pelvic tilt taping for the treatment of chronic low back pain with sacroiliac joint dysfunction and increased sacral horizontal angle. Phys. Ther. Sport.

[29] Lee J.-H., Yoo W.-G., Kim M.-H., Oh J.-S., Lee K.-S., Han J.-T. (2014). Effect of posterior pelvic tilt taping in women with sacroiliac joint pain during active straight leg raising who habitually wore high-heeled shoes: A preliminary study. J. Manip. Physiol. Ther..

[30] Shin Y.-J., Choi E.-H., Choe Y.-W., Peng C., Kim M.-K. (2017). Immediate Effects of Posterior Pelvic Tilting Taping on Gait Ability of Chronic Stroke Patients: A Randomized Controlled Trial. J. Exp. Stroke. Transl. Med..

[31] Güp A.A., Bayar B. (2023). Immediate effects of trunk Kinesio Taping® on functional parameters in the acute stage of patients with mild stroke: A randomized controlled trial. Physiother. Theory Pract..

[32] Bridges T., Bridges C. (2016). Length, Strength and Kinesio Tape-eBook: Muscle Testing and Taping Interventions.

[33] Bozorgmehr A., Takamjani I.E., Akbari M., Salehi R., Mohsenifar H., Rasouli O. (2020). Effect of posterior pelvic tilt taping on abdominal muscle thickness and lumbar lordosis in individuals with chronic low back pain and hyperlordosis: A single-group, repeated-measures trial. J. Chiropr. Med..

[34] Lewis C.L., Laudicina N.M., Khuu A., Loverro K.L. (2017). The human pelvis: Variation in structure and function during gait. Anat. Rec..

[35] Jung K.-S., Jung J.-H., In T.-S., Cho H.-Y. (2022). Effects of Pelvic Stabilization Training with Lateral and Posterior Tilt Taping on Pelvic Inclination, Muscle Strength, and Gait Function in Patients with Stroke: A Randomized Controlled Study. BioMed Res. Int..

[36] Richardson J.T. (2011). Eta squared and partial eta squared as measures of effect size in educational research. Educ. Res. Rev..

[37] Kase K., Wallis J., Kase T. (2003). Clinical therapeutic applications of the Kinesio (! R) taping method.

[38] Pourahmadi M.R., Bagheri R., Jannati E., Takamjani I.E., Sarrafzadeh J., Mohsenifar H. (2018). Effect of elastic therapeutic taping on abdominal muscle endurance in patients with chronic nonspecific low back pain: A randomized, controlled, single-blind, crossover trial. J. Manip. Physiol. Ther..

[39] Bhutto M., Shadmehr A., Hadian M.R., Talebian S., Rana Z., Asad S.A. (2021). Test-retests reliability of digital photography in measuring quadriceps-angle and pelvic tilt angle in healthy population. Pak. J. Med. Health Sci..

[40] Ashnagar Z., Hadian M.R., Olyaei G., Moghadam S.T., Rezasoltani A., Saeedi H., Yekaninejad M.S., Mahmoodi R. (2017). Reliability of digital photography for assessing lower extremity alignment in individuals with flatfeet and normal feet types. J. Bodyw. Mov. Ther..

[41] Verheyden G., Nieuwboer A., Mertin J., Preger R., Kiekens C., De Weerdt W. (2004). The Trunk Impairment Scale: A new tool to measure motor impairment of the trunk after stroke. Clin. Rehabil..

[42] Ko J., You Y. (2015). Reliability and responsiveness of the Korean version of the trunk impairment scale for stroke patients. J. Korean Phys. Ther..

[43] Swanenburg J., de Bruin E.D., Favero K., Uebelhart D., Mulder T. (2008). The reliability of postural balance measures in single and dual tasking in elderly fallers and non-fallers. BMC Musculoskelet. Disord..

[44] Oliveira L.F., Simpson D.M., Nadal J. (1996). Calculation of area of stabilometric signals using principal component analysis. Physiol. Meas..

[45] Palazzo F., Caronti A., Lebone P., Proietti A., Panzarino M., Annino G. (2015). Effects of stimulating surface during static upright posture in the elderly. Somatosens. Mot. Res..

[46] Wong J.S., Jasani H., Poon V., Inness E.L., McIlroy W.E., Mansfield A. (2014). Inter-and intra-rater reliability of the GAITRite system among individuals with sub-acute stroke. Gait Posture.

[47] Hsu A.-L., Tang P.-F., Jan M.-H. (2003). Analysis of impairments influencing gait velocity and asymmetry of hemiplegic patients after mild to moderate stroke. Arch. Phys. Med. Rehabil..

[48] Paoloni M., Bernetti A., Fratocchi G., Mangone M., Parrinello L., Del Pilar Cooper M., Sesto L., Di Sante L., Santilli V. (2011). Kinesio Taping applied to lumbar muscles influences clinical and electromyographic characteristics in chronic low back pain patients. Eur. J. Phys. Rehabil. Med..

[49] Jaron A., Konkol B., Gabrysz-Trybek E., Bladowska J., Grzywacz A., Nedjat A., Trybek G. (2021). Kinesio taping–a healing and supportive method in various fields of medicine, dentistry, sport and physiotherapy. Balt. J. Health Phys. Act..

[50] Gómez-Soriano J., Abián-Vicén J., Aparicio-García C., Ruiz-Lázaro P., Simón-Martínez C., Bravo-Esteban E., Fernández-Rodríguez J.M. (2014). The effects of Kinesio taping on muscle tone in healthy subjects: A double-blind, placebo-controlled crossover trial. Man. Ther..

[51] Tahmasbi A., Soleimani A., Ghotbi N., Malmir K., Shadmehr A. (2022). Effects of Kinesio Taping over Abdominal Muscles with Different Tensions on the Lumbopelvic Complex Components in Men with Increased Anterior Pelvic Tilt. J. Mod. Rehabil..

[52] Liao L.-Y., He X.-H., Li X.-Z., Ge Y.-L., Gao Q. (2020). Effects of kinesiology taping on trunk function, balance, and mobility in stroke patients: A pilot feasibility study. J. Phys. Ther. Sci..

[53] Tanoori P., Mohamed M.N.A., Ali M.R.M. (2016). Effects of kinesio® tape vs rigid tape on shoulder muscle strength in healthy tennis players. Int. J. Appl. Exerc. Physiol..

[54] Levine D., Whittle M.W. (1996). The effects of pelvic movement on lumbar lordosis in the standing position. J. Orthop. Sports Phys. Ther..

[55] Verheyden G., Vereeck L., Truijen S., Troch M., Herregodts I., Lafosse C., Nieuwboer A., De Weerdt W. (2006). Trunk performance after stroke and the relationship with balance, gait and functional ability. Clin. Rehabil..

[56] El-Nabie A., Abd El-Hakiem W., Saleh M.S.M. (2019). Trunk and pelvic alignment in relation to postural control in children with cerebral palsy. J. Back Musculoskelet. Rehabil..

[57] Balaban B., Tok F. (2014). Gait disturbances in patients with stroke. PmR.

[58] Verheyden G., Ruesen C., Gorissen M., Brumby V., Moran R., Burnett M., Ashburn A. (2014). Postural alignment is altered in people with chronic stroke and related to motor and functional performance. J. Neurol. Phys. Ther..

[59] Wang R.Y., Lin C.Y., Chen J.L., Lee C.S., Chen Y.J., Yang Y.R. (2022). Adjunct non-elastic hip taping improves gait stability in cane-assisted individuals with chronic stroke: A randomized controlled trial. J. Clin. Med..

[60] Patterson K.K., Gage W.H., Brooks D., Black S.E., McIlroy W.E. (2010). Evaluation of gait symmetry after stroke: A comparison of current methods and recommendations for standardization. Gait Posture.

[61] In T.-S., Jung J.-H., Kim M., Jung K.-S., Cho H.-Y. (2021). Effect of posterior pelvic tilt taping on pelvic inclination, muscle strength, and gait ability in stroke patients: A randomized controlled study. J. Clin. Med..

[62] Patterson K.K., Parafianowicz I., Danells C.J., Closson V., Verrier M.C., Staines W.R., Black S.E., McIlroy W.E. (2008). Gait asymmetry in community-ambulating stroke survivors. Arch. Phys. Med. Rehabil..

[63] Roerdink M., Beek P.J. (2011). Understanding inconsistent step-length asymmetries across hemiplegic stroke patients: Impairments and compensatory gait. Neurorehabilit. Neural Repair.

[64] Balasubramanian C.K., Bowden M.G., Neptune R.R., Kautz S.A. (2007). Relationship between step length asymmetry and walking performance in subjects with chronic hemiparesis. Arch. Phys. Med. Rehabil..

